# Supplementary material for: Heat adaptation of phage T7 under an extended genetic code
Source: Virus Evol. 2021 Dec 1;7(2):veab100. doi: 10.1093/ve/veab100 (PMC8923235; doi:10.1093/ve/veab100)
Supplement: veab100_Supp [file veab100_supp.zip › Sequencing_Analysis.zip!/Sequencing_Analysis/S2/evidence/RA_32.html]

BRESEQ :: Evidence


| Read alignment evidence... | | | | | | | | | | | |
| --- | --- | --- | --- | --- | --- | --- | --- | --- | --- | --- | --- |
|  | seq id | position | | ref | new | freq | score (cons/poly) | reads | annotation | genes | product |
| \* | NC\_001604.1 | 35,254 | 0 | A | G | 37.3% | 3426.1 / inf | 4876 | intergenic (–/–) | *–/–* | –/– |
| Reads supporting (aligned to +/- strand):  **ref** base A (1543/1498);  **new** base G (941/865);  **total** (2504/2372) | | | | | | | | | | | |
| Fisher's exact test for biased strand distribution *p*-value = 3.73e-01 | | | | | | | | | | | |
| Kolmogorov-Smirnov test that lower quality scores support variant *p*-value = 9.85e-01 | | | | | | | | | | | |

Only 100 of 4893 total aligned reads displayed.  

`GAAGAGGCCCGTGACCTCACTACGGATACTATCGGTGTCAATAACGATGGTCACTTGGATGCTCGTGGTCGTCGAATTGTGAACCTAGCGAACGCCGTGGATGACCGCGATGCT‑GTTCCGTTTGGTCAACTAAAGACCATGAACCAGAACTCATGGCAAGCACGTAATGAAGCCTTACAGTTCCGTAATGAGGCTGAGACTTTCAGAAACCAAGC‑GGAGGGCTTTAAGAACGAGTCCAGTACCAACGCTAC‑GAACACAAAGCAGTGGCGCGATGAGACCAAGGGTTTCCGAGACGAAGCCAAGCGGTTCAAGAATACGGCTGGTCAAT‑ACGCTACATCTGCTGGG‑AACTCTGCTTCCGCTGCGCATCAATCTGAGGTAAACGCTGAGAACTCTGCCACAGCATCCGCTAACTCTGCTCATTTGGCAGAACAGCAAGCAGACCGTGCGGAACGTGAGGCAGACAAGCTGGAAAATTACAATGGATTGGCTGGTGCAATTGATAAGGTAGATGGAACCAATGTGTACTGGAAAGGAAATATTCACGCTAACGGGCGCCTTTACATGACCACAAACGGTTTTGACTGTGGCCAGTATCAACAGT  >  NC_001604.1/34954‑35554`  

                                                                                                                                                                                                                                                                                                               `|`                                                                                                                                                                                                                                                                                                                
    `gggCCCGTGACCTCACTGCGGATACTATCGGTGTCAATAACGATGGTCACTTGGATGCTCGTGGTCGTCGAATTGTGAACCTAGCGAACGCCGTGGATGACCGCGATGCT‑GTTCCGTTTGGTCAACTAAAGACCATGGAACAGAACTCATGGCAAGCACGTAATGAAGCCTTACAGTTCCGTAATGAGGCTGAGACTTTCAGAAACCAAGC‑GGAGGGCTTTAAGAACGAGTCCAGTACCAACGCTAC‑GAACACAAAGCAGTGGCGCGATGAGACCAAGGGTTTCCGAGACGAAGCCAAGCg                                                                                                                                                                                                                                                                                                            <  1:716037/300‑1 (MQ=255)`  
             `cccTCACTGCCGTTACTATCGTTGTCAATAACGATGGTCACTTGGATGCTCGTGGTCGTCGAATTGTGAACCTAGCGAACGCCGTGGATGACCGCGATGCT‑GTTCCGTTTGGTCAACTAAAGACCATGGACCAGAACTCATGGCAAGCACGTAATGAAGCCTTACAGTTCCGTAATGAGGCTGAGACTTTCAGAAACCAAGC‑GGAGGGCTTTAAGAACGAGTCCAGTACCAACGCTAC‑GAACACAAAGCAGTGGCGCGATGAGACCAAGGGTTTCCGAGACGAAGCCGAGCGGTTCAAGaa                                                                                                                                                                                                                                                                                                   <  1:809590/300‑1 (MQ=255)`  
             `aCCTCACTGCGGATACTATCGGTGTCAATAACGATGGTCACTTGGATGCTCGTGGTCGTCGAATTGTGAACCTAGCGAACGCCGTGGATTACCGCGATGCT‑GTTCCGTTTGGTCAACTAAAGACCATGGACCAGAACTCATGGCAAGCACGTAATGAAGCCTTACAGTTCCGTAATGAGGCTGAGACTTTCAGAAACCAAGC‑GGAGGGCTTTAAGAACGAGTCCAGTACCAACGCTAC‑GAACACAAAGCAGTGGCGCGATGAGACCAAGGGTTTCCGAGACGAAGCCAAGCGGTTCAAGaa                                                                                                                                                                                                                                                                                                   <  1:52069/301‑1 (MQ=255)`  
             `aCCTCACTGCGGATACTATCGGTGTCAATAACGATGGTCACTTGGATGCTCGTGGTCGTCGAATTGTGAACCTAGAGAACGCCGTGGATGACCGCGATGCT‑GTTCCGTTTGGTCAACTAAAGACCATGGACCAGAACTCATGGCAAGCACGTAATGAAGCCTTACAGTTCCGTAATGAGGCTGAGACTTTCAGAAACCAAGC‑GGAGGGCTTTAAGAACGAGTCCAGTACCAACGCTAC‑GAACACAAAGCAGTGGCGCGATGAGACCAAGGGTTTCCGAGACGAAGCCAAGCGGTTCAAGaa                                                                                                                                                                                                                                                                                                   <  1:250861/301‑1 (MQ=255)`  
              `ccTCACTGCGGATACTATCGGTGTCAATAACGATGGTCACTTGGATGCTCGTGCTCGTCGAATTGTGAACCTAGCGAACGCCGTGGATGACCGCGATGCT‑TTTCCGTTTGGTCAACTAAAGACCATGGCCCAGAACTAATGGCAAGCACGTTATGAAGCCTTACAGTTCCGTAATGAGGCTGAGACTTTCAGAAACCAAGC‑GGTGGGCTTTAAGAACGAGTCCAGTACCAACGCTAC‑GAATACAAAGCAGTGGCCAGATGAGACCAATGTTTTCCGAGACGAAGCCAAACGGTTTTAGAAt                                                                                                                                                                                                                                                                                                  >  1:544887/1‑301 (MQ=255)`  
                   `ctgcGGATACTATCGGTGTCAATAACGATGGTCACTTGGATGCTCGTGGTCGTCGAATTGTGAACCTAGCGAACGCCGTGGATGACCGCGATGCT‑GTTCCGTTTGGTCAACTAAAGACCATGGACCAGAACTCATGGCAAGCACGTAATGAAGCCTTACAGTTCCGTAATGAGGCTGAGACTTTCAGAAACCAAGC‑GGAGGGCTTTAAGAACGAGTCCAGTACCAACGCTAC‑GAACACAAAGCAGTGGCGCGATGAGACCAAGGGTTTCCGAGACGAAGCCAAGCGGTTCAAGAATACGGc                                                                                                                                                                                                                                                                                             <  1:307042/298‑1 (MQ=255)`  
                   `ctgcGGATACTATCGGTGTCAATAACGATGGTCACTTGGATGCTAGTGGTCGTCGAATTGTGAACCTAGCGAACGCCGTGGATGACCGCGATGCT‑GTTCCGTTTGGTCAACTAAAGACCATGGACCAGAACTCATGGCAAGCACGTAATGAAGCCTTACAGTTCCGTAATGAGGCTGAGACTTTCAGAAACCAAGC‑GGAGGGCTTTAAGAACGAGTCCAGTACCAACGCTAC‑GAACACAAAGCAGTGGCGCGATGAGACCAAGGGTTTCCGAGACGAAGCCAAGCGGTTCAAGAATACGGc                                                                                                                                                                                                                                                                                             <  1:777923/298‑1 (MQ=255)`  
                      `cGGATACTATCGGTGTCAATAACGATGGTCACTTGGATGCTCGTGGTCGTCGAATTGTGAACCTAGCGAACGCCGTGGATGACCGCGATGCT‑GTTCCGTTTGGTCAACTAAAGACCATGGACCAGAACTCATGGCAAGCATGTAATGAAGCCTTACAGTTCCGTAATGAGGCTGAGACTTTCAGAAACCAAGC‑GGAGGGCTTTAAGAACGAGTCCAGTACCAACGCTAC‑GAACACAAAGCAGTGGCGCGATGAGACCAAGGGTTTCCGAGACGAAGCCAAGCGGTTCAAGAATACGGcctg                                                                                                                                                                                                                                                                                          >  1:139405/1‑298 (MQ=255)`  
                       `ggATACTATCGGTGTCAATAACGATGGTCACTTGGATGCTCGTGGTCGTCGAATTGTGAACCTAGCGAACGCCGTGGATGACCGCGATGCT‑GTTCCGTTTGGTCAACTAAAGACCATGGACCAGAACTCATGGCAAGCACGTAATGAAGCCTTACAGTTCCGTAATGAGGCTGAGACTTTCAGAAACCAAGC‑GGAGGGCTTTAAGAACGAGTCCAGTACCAACGCTAC‑GAACACAAAGCAGTGGCGCGATGAGACCAAGGGTTTCCGAGACGAAGCCGAGCGGTTCCAGAATACGGCTGGt                                                                                                                                                                                                                                                                                         >  1:504318/1‑301 (MQ=255)`  
                       `ggATACTATCGGTGTCAATAACGATGGTCACTTGGATGCTCGTGGTCGTCGAATTGTGAACCTAGCGAACGCCGTGGATGACCGCGATGCT‑GTTCCGTTTGGTCAACTAAAGACCATGGACCAGAACTCATGGCAAGCACGTAATGAAGCCTTACAGTTCCGTAATGAGGCTGAGACTTTCAGAAACCAAGC‑GGAGGGCTTTAAGAACGAGTCCAGTACCAACGCTAC‑GAACACAAAGCAGTGGCGCGATGAGACCAAGGGTTTCCGAGACGAAGCCAAGCGGTTCAAGAATACGGCTggg                                                                                                                                                                                                                                                                                         >  1:23007/1‑300 (MQ=255)`  
                          `tACTATCGGTGTCAATAACGATGGTCACTTGGATGCTCGTGGTCGTCGAATTGTGAACCTAGCTAACGCCGTGGATGACCGCGATGCT‑GTTCCGTTTGGTCAACTAAAGACCATGGACCAGAACTCATGGCAAGCACGTAATGAAGCCTTACAGTTCCGTAATGAGGCTGAGACTTTCAGAAACCAAGC‑GGAGGGCTTTAAGAACGAGTCCAGTACCAACGCTAC‑GAACACAAAGCAGTGGCGCGATGAGACCAAGGGTTTCCGAGACGAAGCCAAGCGGTTCAAGAATACGGCTGGTCaa                                                                                                                                                                                                                                                                                      >  1:314792/1‑301 (MQ=255)`  
                          `tACTATCGGTGTCAATAACGATGGTCACTTGGATGCTCGTGGTCGTCGAATTGTGAACCTAGCGAACGCCGTGGATGACCGCGATGCT‑GTTCCGTTTGGTCAACTAAAGACCATGGACCAGAACTCATGGCAAGCACGTAATGAAGCCTTACAGTTCCGTAATGAGGCTGAGACTTTCAGAAACCAAGC‑GGAGGGCTTTAAGAACGAGTCCAGTACCAACGCTAC‑GAACACAAAGCAGTGGCGCGATGAGACCAAGGGTTTCCGAGACGAAGCCGAGCGGTTCAAGAATACGGCTGGTCaa                                                                                                                                                                                                                                                                                      >  1:285122/1‑301 (MQ=255)`  
                             `aagagacagcAATAACGATGGTCACTTGGAGGCTCGTGGTCGTCGAATGGTGAACCTAGCGAACGCCGTGGATGACCGCGATGCT‑GTTCCGTTTGGTAAACTAAAGACCATGGACCAGAACTCATGGCAAGCACGTAATGAAGCCTTACAGTTCCGTAATGAGGCTGAGACTTTCAGAAACCAAGC‑GGAGGGCTTTAAGAACGAGTCCAGTACCAACGCTAC‑GAACACAAAGCAGTGGCGCGATGAGACCAAGGGTTTCCGAGACGAAGCCAAGCGGTTCAAGAATACGGCTGGTCAAT‑Ac                                                                                                                                                                                                                                                                                  <  1:83723/292‑1 (MQ=255)`  
                                    `gtCAATAACGATGGTCACTTGGATGCTCGTGGTCGTCGAATTGTGAACCTAGCGAACGCCGTGGATGACCGCGATGCT‑GTTCCGTTTGGTCAACTAAAGACCATGGACCAGAACTCATGGCAAGCACGTAATGAAGCCTTACAGTTCCGTAATGAGGCTGAGACTTTCAGAAACCAAGC‑GGAGGGCTTTAAGAACGAGTCCAGTACCAACGCTAC‑GAACACAAAGCAGTGGCGCGATGAGACCAAGGGTTTCCGAGACGAAGCCGAGCGGTTCAAGAATACGGCTGGTCAAT‑ACGCTACAt                                                                                                                                                                                                                                                                           >  1:529496/1‑301 (MQ=255)`  
                                    `gtCAATAACGATGGTCACTTGGATGCTCGTGGTCGTCGAATTGTGAACCTAGCGAACGCCGTGGATGACCGCGATGCT‑GTTCCGTTTGGTCAACTAAAGACCATGGACCAGAACTCATGGCAAGCACGTAATGAAGCCTTACAGTTCCGTAATGAGGCTGAGACTTTCAGAAACCAAGC‑GGAGGGCTTTAAGAACGAGTCCAGTACCAACGCTAC‑GAACACAAAGCAGTGGCGCGATGAGACCAAGGGTTTCCGAGACGAAGCCAAGCGGTTCAAGAATACGGCTggctgtctcttataca                                                                                                                                                                                                                                                                            >  1:420127/1‑287 (MQ=255)`  
                                     `tCAATAACGATGGTCACTTGGATGCTCGTGGTCGTCGAATTGTGAACCTAGCGAACGCCGTGGATGACCGCGATGCT‑GTTCCGTTTGGTCAACTAAAGACCATGGACCAGAACTCATGGCAAGCACGTAATGAAGCCTTACAGTTCCGTAATGAGGCTGAGACTTTCAGAAACCAAGC‑GGAGGGCTTTAAGAACGAGTCCAGTACCAACGCTAC‑GAACACAAAGCAGTGGCGCGATGAGACCAAGGGTTTCCGAGACGAAGCCGAGCGGTTCAAGAATACGGCTGGTCAAT‑ACGCTACATc                                                                                                                                                                                                                                                                          <  1:270450/301‑1 (MQ=255)`  
                                                `ggTCACTTGGATGCTCGTGGTCGTCGAATTGTGAACCTAGCGAACGCCGTGGATGACCGCGATGCT‑GTTCCGTTTGGTCAACTAAAGACCATGGACCAGAACTCATGGCAAGCACGTAATGAAGCCTTACAGTTCCGTAATGAGGCTGAGACTTTCAGAAACCAAGC‑GGAGGGCTTTAAGAACGAGTTCAGTACCAACGCTAC‑GAACACAAAGCAGTGGCGCGATGAGACCAAGGGTTTCCGAGACGAAGCCGAGCGGTTCAAGAATACGGCTGGTCAAT‑ACGCTACATCTGCTGGG‑CAct                                                                                                                                                                                                                                                              >  1:29930/1‑301 (MQ=255)`  
                                                 `gTCACTTGGATGCTCGTGGTCGTCGAATTGTGAACCTAGCGAACGCCGTGGATGACCGCGATGCT‑GTTCCGTTTGGTCAACTAAAGACCATGGACCAGAACTCATGGCAAGCACGTAATGAAGCCTTACAGTTCCGTAATGAGGCTGAGACTTTCAGAAACCAAGC‑GGAGGGCTTTAAGAACGAGTCCAGTACCAACGCTAC‑GAACACAAAGCAGTGGCGCGATGAGACCAAGGGTTTCCGAGACGAAGCCAAGCGGTTCAAGAATACGGCTGGTCAAT‑ACGCTACATCTGCTGGG‑AActc                                                                                                                                                                                                                                                             <  1:710743/301‑1 (MQ=255)`  
                                                        `ggATGCTCGTGGTCGTCGAATTGTGAACCTAGCGAACGCCGTGGATGACCGCGATGCT‑GTTCCGTTTGGTCAACTAAAGACCATGGACCAGAACTCATGGCAAGCACGTAATGAAGCCTTACAGTTCCGTAATGAGGCTGAGACTTTCAGAAACCAAGC‑GGAGGGCTTTAAGAACGAGTCCAGTACCAACGCTAC‑GAACACAAAGCAGTGGCGCGATGAGACAAAGGGTTTCCGAGACGAAGCCAAGCGGTTCAAGCATACGGCTGGTCAAT‑ACGCTACATCTGCTGGG‑AACTCTACTTcc                                                                                                                                                                                                                                                      >  1:390367/1‑301 (MQ=255)`  
                                                          `aTGCTCGTGGTCGGCGAATTGTGAACCTAGCGAACGCCGTGGATGACCGCGATGCT‑GTTCCGTTTGGTCAACTAAAGACTATGGACCAGAACTCATGGCAAGCACGTAATGAAGCCTTACAGTTCCGTAATGAGGCTGAGACTTTCAGAAACCAAGC‑GGAGGGCTTTAAGAACGAGTCCAGTACCAACGCTAC‑GAACACAAAGCAGTGGCGCGATGAGACCAAGGGTTTCCGAGACGAAGCCAAGCGGTTCAAGAATACGGCTGGTCAAT‑ACGCTACATCTGCTGGG‑AACTCTGCTTCCGc                                                                                                                                                                                                                                                    <  1:107164/301‑1 (MQ=255)`  
                                                                    `tcgttgAATTGTGAACCTAGCGAACGCCGTGGATGTCCGCGATGCT‑GTTCCGTTTGGTCAACTAAAGACCATGGACCAGAACTCATGGCAAGCACGTAATGAAGCCTTACAGTTCCGTAATGAGGCTGAGACTTTCAGAAACCAAGC‑GGAGGGCTTTAAGAACGAGTCCAGTACCAACGCTAC‑GAACACAAAGCAGTGGCGCGATGAGACCAAGGGTTTCCGAGACGAAGCCAAGCGGTTCAAGAATATGGCTGGTCAAT‑ACGCTACATCTGCTGGG‑AACTCTGCTTCCGCTGCGCATCaa                                                                                                                                                                                                                                          >  1:799331/1‑301 (MQ=255)`  
                                                                       `gcgAATTGTGAAACTGGCGAACGCCGTGGAGTACCGCGATGCT‑GTTCCGTTTGGACAACTAAAGACCATGGACCAGAACTCATGGCAAGCACGTAATGAAGCCTTACAGTTCCGTAATGAGGCTGAGACTTTCAGAAACCAAGC‑GGAGATCTTTAAGAACGAGTCCAGTACCAACGCTAC‑GAACACAAAGCAGTGGCGCGATGAGACCAAGGGTTTCCGAGACGAAGCCGATCGGTTCAAGAATACGGCTGGTCAAT‑ACGCTACATCTGCTGGG‑AACTCTGCTTCCGCTGCGCATCAATCt                                                                                                                                                                                                                                       <  1:159612/300‑1 (MQ=255)`  
                                                                           `aTTGTGAACCTAGCGAACGCCGTGGATGACCGCGATGCT‑GTTCCGTTTGGTCAACTAAAGACCATGGACCAGAACTCATGGCAAGCACGTAATGAAGCCTT‑CCGGTCCCTAATGAAGGTTAGACTTTCAGAAACCAAGCGGGAGGGCTTAAGAAACAGGCCCAGTACAAAC‑CTAC‑GAAACCAAAGCAATGGCGCGAAGAGAACAAAGGGTTCCGAGACCAAGCCCGACGGGTCAAGAATAAGGCTGGGTAATGACG‑TAACTCTGCTGGGAAACTC‑GCTTTCGCCGCCCATCTAACGGAggg                                                                                                                                                                                                                                  >  1:292512/1‑300 (MQ=255)`  
                                                                           `aTTGTGAACCTAGCGAACGCCGTGGATGACCGCGATGCT‑GTTCCGTTTGGTCAACTAAAGACCATGGACCAGAACTCATGGCAAGCACGTAATGAAACCTTACAGTTCCGTAATGAGGCTGAGACTTTCAGAAACCAAGT‑GGAGGGCTTTAAGAACGAGTCCAGTAACAACGTTAC‑GAACAGAAAGCAGTGGAGCGATGAGACCAAGGGCTTTCGTGACGATGCCAAGCGGTTCAAGAATACTGCTGGTCAAT‑ACGCTACATCTGCTGGG‑AACTCTGCTTCCGCTGCGCATCAATTTGAgg                                                                                                                                                                                                                                   >  1:746149/1‑301 (MQ=255)`  
                                                                                `gagccTAGCGGACGCCGTGGAGGAGCGCGATGGT‑GTTCCGTTTGGTCAACTAAAGACCATGGACGAGAACTCAAGGCAAGCACATAATGAAGCCTTACAGTTCCGTAAGGAGGCTGAGACTTTCAGATACCGAGC‑GAGGGGCTTTAAAAACGAGTACAGTACCAAAGCTAC‑GAACAGAAAGCAGTGGCGCGATGGGACCAGGGGTTTCCGAGACGAAGCCGAGCGGTTCAAGAATACGGCTGGTCAAT‑ACGCTACATCTGCTGGG‑AACACTGCTTCCGCTGCGCATCAATCTGAGGTAAAc                                                                                                                                                                                                                              <  1:429852/298‑1 (MQ=255)`  
                                                                                `gAACCTAGCGAACGCCGTGGATGACCGCGATGCT‑GTTCCGTTTGGTCAACTAAAGACCATGGACCAGAACTCATGGCAAGCACGTAATGAAGCCTTACAGTTCCGTAATGAGGCTGAGACTTTCAGAAACCAAGC‑GGAGGGCTTTAAGAACGAGTCCAGTACCAACGCTAC‑GAACACAAAGCAGTGGCGCGATGAGACCAAGGGTTTCCGAGACGAAGCCAAGCGGTTCAAGAATATGGCTGGTCAAT‑ACGCTACATCTGCTGGG‑AACTCTGCTTCCGCTGCGCATCAATCTGAGGTAAAc                                                                                                                                                                                                                              <  1:69580/301‑1 (MQ=255)`  
                                                                                  `aCCTAGCGAACGCCGTGGATGACCGCGATGCT‑GTTCCGTTTGGTCAACTAAAGACCATGGACCAGAACTCATGGCAAGCACGTAATGAAGCCTTACAGTTCCGTAATGAGGCTGAGACTTTCAGAAACCAAGC‑GGAGGGCTTTAAGAACGAGTCCAGTACCAACGCTAC‑GAACACAAAGCAGTGGCGCGATGAGACCAAGGGTTTCCGAGACGAAGCCAAGCGGTTCAAGAATACGGCTGGTCAAT‑ACGCTACATCTGCTGGG‑AACTCTGCTTCCGATGCGCATCAATCTGAGGTAAACGc                                                                                                                                                                                                                            <  1:583063/301‑1 (MQ=255)`  
                                                                                   `ccTAGCGAACGCCGTGGATGACCGCGATGCT‑GTTCCGTTTGGTCAACTAAAGACCATGGACCAGAACTCATGGCAAGCACGTAATGAAGCCTTACAGTTCCGTAATGAGGCTGAGACTTTCAGAAACCAAGC‑GGAGGGCTTTAAGAACGAGTCCAGTACCAACGCTAC‑GAACACAAAGCAGTGGCGCGATGAGACCAAGGGTTTCCGAGACGAAGCCAAGCGGTTCAAGAATACGGCTGGTCAAT‑ACGCTACATCTGCTGGG‑AACTCTGCTTCCGCTGCGCATCAATCTGAGGTAAACGCt                                                                                                                                                                                                                           >  1:33845/1‑301 (MQ=255)`  
                                                                                     `tAGCGAACGCCGTGGATGACCGCGATGCT‑GTTCCGTTTGGTCAACTAAAGACCATGGACCAGAACTCATGGCAAGCACGTAATGAAGCCTTACAGTTCCGTAATGAGGCTGAGACTTTCAGAAACCAAGC‑GGAGGGCTTTAAGAACGAGTCCAGTACCAACGCTAC‑GAACACAAAGCAGTGGCGCGATGAGACCAAGGGTTTCCGAGACGAAGCCGAGCGGTTCAAGAATACGGCTGGTCAAT‑ACGCTACATCTGCTGGG‑AACTCTGCTTCCGCTGCGCATCAATCTGAGGTAAACGCTgg                                                                                                                                                                                                                         >  1:382682/1‑300 (MQ=255)`  
                                                                                      `aGCGAACGCCGTGGATGACCGCGATGCT‑GTTCCGTTTGGTCAACTAAAGACCTTGGACCAGAACTCATGGCAAGCACGTAATGAAGCCTTACAGTTCCGTAATGAGGCTGAGACTTTCAGAAACCAAGC‑GGAGGGCTTTAAGAACGAGTCCAGTACCAACGCTAC‑GAACACAAAGCAGTGGCGCGATGAGACCAAGGGTTTCCGAGACGAAGCCAAGCGGTTCAAGAATACGGCTGGTCAAT‑ACGCTACATCTGCTGGG‑AACTCTGCTTCCGCTGCGCATCAATCTGAGGTAAACGCTgag                                                                                                                                                                                                                        >  1:244940/1‑301 (MQ=255)`  
                                                                                         `tgtataagagaaagACCGCGATGCT‑GTTCCGTTTGGTCAACTAAAGACCATGGACCAGAACTCATGGCAAGCACGTAATGAAGCCTTACAGTTCCGTAATGAGGCTGAGACTTTCAGAAACCAAGC‑GGAGGGCTTTAAGAACGAGTCCAGTACCAACGCTAC‑GAACACAAAGCAGTGGCGCGATGAGACCAAGGGTTTCCGAGACGAAGCCAAGCGGTTCAAGAATACGGCTGGTCAAT‑ACGCTACATCTGCTGGG‑AACTCTGCTTCCGCTGCGCATCAATCTGAGGTAAACGCTGAGAAc                                                                                                                                                                                                                     <  1:91220/288‑1 (MQ=255)`  
                                                                                         `ggacccccgggATGGCCGCGAAGGTGGTTCCGTTGGGTAAACTAAAGACAAGGGCCAAAAAACAAGGGAAAGCACGAAATGAAGCTTAAAAGTCCCGAAAGGGGGTTGAGATTTTCAGAAACAAAGC‑GGAGGGTTTAAAGAACAAGCCCAGT‑CCAACCGTACGGAACAAAAAGAGGGGGGGGAAGGGGACCAAGGGTTCCCGAGACAAAGCAAAGCGGTTCAAG‑ATTCGGGTGGGCAAA‑AAGGTTAAACCGGCGGG‑AAACCTGGTTCCGCTGCGCATCAATCTGAGGTAAACGCTGAGAAc                                                                                                                                                                                                                     <  1:11308/292‑1 (MQ=255)`  
                                                                                           `aCGCCGTGGATGACCGCGATGCT‑GTTCCGTTTGGTCAACTAAAGACCATGGACCAGAACTCATGGCAAGCACGTAATGAAGCCTTACAGTTCCGTAATGAGGCTGAGACTTTCAGAAACCAAGC‑GGAGGGCTTTAAGAACGAGTCCAGTACCAACGCTAC‑GAACACAAAGCAGTGGCGCGATGAGACCAAGGGTTTCCGAGACGAAGCCAAGCGGTTCAAGAATACGGCTGGTCAAT‑ACGCTACATCTGCTGGG‑AACTCTGCTTCCGCTGCGCATCAATCTGAGGTAAACGCTGAGAActc                                                                                                                                                                                                                   <  1:648723/301‑1 (MQ=255)`  
                                                                                            `cGCCGTGGATGACCGCGATGCT‑GTTCCGTTTGGTCAACTAAAGACCATGGACCAGAACTCATGGCAAGCACGTAATGAAGCCTTACAGTTCCGTAATGAGGCTGAGACTTTCAGAAACCAAGC‑GGAGGGCTTTAAGAACGAGTCCAGTACCAACGCTAC‑GAACACAAAGCAGTGGCGCGATGAGACCAAGGGTTTCCGAGACGAAGCCGAGCGGTTCAAGAATACGGCTGGTCAAT‑ACGCTACATCTGCTGGG‑AACTCTGCTTCCGCTGCGCATCAATCTGAGGTAAACGCTGAGAActct                                                                                                                                                                                                                  >  1:359281/1‑301 (MQ=255)`  
                                                                                                   `gATGACCTCGATGCT‑GTTCCGTTTGGTCAACTAAAGACCATGGACCAGAACTCATGGCAAGCACGTAATGAAGCCTTACAGTTCCGTAATGAGGCTGAGACTTTCAGAAACCAAGC‑GGAGGGCTTTAAGAACGAGTCCAGTACCAACGCTAC‑GAACACAAAGCAGTGGCGCGATGAGACCAAGGGTTTCCGAGACGAAGCCAAGCGGTTCAAGAATACGGCTGGTCAAT‑ACGCTACATCTGCTGCG‑AACTCTGCTTCCGCTGCGCATCAATCTGAGGTAAACGCTGAGAACTCTGCCACAg                                                                                                                                                                                                           <  1:237964/301‑1 (MQ=255)`  
                                                                                                               `gCT‑GTTCCGTTTGGTCAACTAAAGACCATGGACCAGAACTCATGGCAAGCACGTAATGAAGCCTTACAGTTCCGTAATGAGGCTGAGACTTTCAGAAACCAAGC‑GGAGGGCTTTAAGAACGAGTCCAGTACCAACGCTAC‑GAACACAAAGCAGTGGCGCGCTGAGACCAAGGGTTTCCGAGACGAAGCCGAGCGGTTCAAGAATACGGCTGGTCAAT‑ACGCTCCATCTGCTGGG‑AACTCTGCTTCCGCTGCGCATCAATCTGAGGTAAACGCTGAGAACTATGccctgtctcttatacaca                                                                                                                                                                                               >  1:792995/1‑285 (MQ=255)`  
                                                                                                                `cT‑GTTCCGTTTGGTCAACTAAAGACCATGGACCAGAACTCATGGCAAGCACGTAATGAAGCCTTACAGTTCCGTAATGAGGCTGAGACTTTCAGAAACCAAGC‑GGAGGGCTTTAAGAACGAGTCCAGTACCAACGCTAC‑GAACACAAAGCAGTGGCGCGATGAGACCAAGGGTTTCCGAGACGAAGCCGAGCGGTTCAAGAATACGGCTGGTCAAT‑ACGCTACATCTGCTGGG‑AACTCTGCTTCCGCTGCGCATCAATCTGAGGTAAACGCTGAGAACTCTGCCACAGCAGCCGCTAActa                                                                                                                                                                                              <  1:762356/301‑2 (MQ=255)`  
                                                                                                                         `tttGGTCAACTAAAGACCAGGGACCAGAACTCATGGAAAGCACGTAATGAAGCCTTACAGTTCCGTAATGAGGCTGAGAATTTCAGAAACCAAGC‑GGAGGGCTTTAAGAACGAGTCCAGTACCAACGCTAC‑GAACACAAAGCAGTGGCGCGATGAGACCAAGGGTTTCCGAGACGAAGCCAAGCGGTTCAAGGATACGGCTGGTCAAT‑ACGCTACATCTGCTGGG‑AACTCTGCTTCCGCTGCGCATCAATCTGAGGTAAACGCTGAGAACTCTGCCACAGCATCCGCTAACTATGCTCAtt                                                                                                                                                                                      <  1:822631/301‑1 (MQ=255)`  
                                                                                                                                `aaCTAAAGACCATGGACCAGAACTCATGGCAAGCACGTAATGAAGCCTTACAGTTCCGTAATGAGGCTGAGACTTTCAGAAACCAAGC‑GGAGGGCTTTAAGAACGAGTCCAGTACCAACGCTAC‑GAACACAAAGCAGTGGCGCGATGAGACCAAGGGTTTCCGAGACGAAGCCAAGCGGTTCAAGAATACGGCTGGTCAAT‑ACGCTACATCTGCTGGG‑AACTCTGCTTCCGCTGCGCATCAATCTGAGGTAAACGCTGAGAACTCTGCCACAGCATCCGCTAACTATGCTCATTTGGCAGa                                                                                                                                                                               <  1:754574/301‑1 (MQ=255)`  
                                                                                                                                 `aCTAAAGACCATGGACCAGAACTCATGGCAAGCACGTAATGAAGCCTTACAGTTCCGTAATGAGGCTGAGACTTTCAGAAACCAAGC‑GGAGGGCTTTAAGAACGAGTCCAGTACCAACGCTAC‑GAACACAAAGCAGTGGCGCGATGAGACCAAGGGTTTCCGAGACGAAGCCAAGCGGTTCAAGAATACGGCTGGTCAAT‑ACGCTACATCTGCTGGG‑AACTCTGCTTCCGCTGCGCATCAATCTGAGGTAAACGCTGAGAACTCTGCCACAGCATCCGCTAACTATGCTCATTTGGCAGaa                                                                                                                                                                              >  1:513010/1‑301 (MQ=255)`  
                                                                                                                                 `aCTAAAGACCATGGACCAGAACTCATGGCAAGCACGTAATGAAGCCTTACAGTTCCGTAATGAGGCTGAGACTTTCAGAAACCAAGC‑GGAGGGCTTTAAGAAAGAGTCCAGTACCAACGCTAC‑GAACACAAAGCAGTGGCGCGATTAGACCAAGGGTTTCCGAGACGACTCCAAGCGGTTCAAGAATAAGGCTGCTCAAT‑ACGCTACATATGCTGGG‑AACTCTCATTCCGCTTCGCATCAAACTCAGGTAAACGCTGAGAACCATGCCCCAGACCCCGCTAACTCTTCACATTTGGCAGaa                                                                                                                                                                              >  1:442320/1‑301 (MQ=255)`  
                                                                                                                                        `aCCATGGACCAGAACTCATGGCAAGCACGTAATGAAGCCTTACAGTTCCGTAATGAGGCTGAGACTTTCAGAAACCAAGC‑GGAGGGCTTTAAGAACGAGTCCAGTACCAACGCTAC‑GAACACAAAGCAGTGGCGCGATGAGACCAAGGGTTTCCGAGACGAAGCCAAGCGGTTCAAGAATACGGCTGGTCAAT‑ACGCTACATCTGCTGGG‑AACTATGCTTCCGCTGCGCATCAATCTGAGGTAAACGCTGAGAACTCTGCCACAGCAGCCGCTAACTCTGCCCATTTGGCAGAACagcaag                                                                                                                                                                       >  1:352315/1‑301 (MQ=255)`  
                                                                                                                                             `ggaCCAGAACTCATGGCAAGCACGTAATGAAGCCTTACAGTTCCGTAATGAGGCTGAGACTTTCAGAAACCAAGC‑GGAGGGCTTTAAGAACGAGTCCAGTACCAACGCTAC‑GAACACAAAGCAGTGGCGCGATGAGACCAAGGGTTTCCGAGACGAAGCCAAGCGGTTCAAGAATACGGCTGGTCAAT‑ACGCTACATCTGCTGGG‑AACTCTGCTTCCGCTGCGCATCAATCTGAGGTAAACGCTGAGAACTCTGCCACAGCAGCCGCTAACTCTGCTCATTTGGCAGAACagcaagcctgt                                                                                                                                                                  >  1:723117/3‑297 (MQ=255)`  
                                                                                                                                             `ggaCCAGAACTCATGGCAAGCACGTAATGAAGCCTTACAGTTCCGTAATGAGGCTGAGACTTTCAGAAACCAAGC‑GGAGGGCTTTAAGAACGAGTCCAGTACCAACGCTAC‑GAACACAAAGCAGTGGCGCGATGAGACCAAGGGTTTCCGAGACGAAGCCAAGCGGTTCAAGAATACGGCTGGGCAAT‑ACGCTACATCTGCTGGG‑AACTCTGCTTCCGCTGCGCATCAATCTGAGGTAAACGCTGAGAAATCTGCCACAGCATCCGCTAACCCTGCTCAGTTGGCAGAAGAGAAAGCAGAc                                                                                                                                                                  >  1:456667/3‑301 (MQ=255)`  
                                                                                                                                                `ccAGAACTCATGGCAAGCACGTAATGAAGCCTTACAGTTCCGTAATGAGGCTGAGACTTTCAGAAACCAAGC‑GGAGGGCTTTAAGAACGAGTCCAGTACCAACGCTAC‑GAACACAAAGCAGTGGCGCGATGAGACCAAGGGTTTCCGAGACGAAGCCAAGCGGTTCAAGAATACGGCTGGTCAAT‑ACGCTACATCTGCTGGG‑AACTCTGCTTCCGATGCGCATCAATCTGAGGTAAACGCTGAGAACTCTGCCACAGCAGCCGCTAACTCTGCTCATTTGGcctgtctcttatacacatct                                                                                                                                                               >  1:559302/1‑282 (MQ=255)`  
                                                                                                                                                   `gAACTCATGGCAAGCACGTAATGAAGCCTTACAGTTCCGTAATGAGGCTGAGACTTTCAGAAACCAAGC‑GGAGGGCTTTAAGAACGAGTCCAGTACCAACGCTAC‑GAACACAAAGCAGTGGCGCGATGAGACCAAGGGTTTCCGAGACGAAGCCAAGCGGTTCAAGAATACGGCTGGTCAAT‑ACGCTACATCTGCTGGG‑TACTCTGCTTCCGCTGCGCATCAATCTGAGGTAAACGCTGAGAACTCTGCCACAGCAGCCGCTAACTCTGCTCATTTGGCAGAACAGCAAGCAGACCGTGCg                                                                                                                                                            >  1:688774/1‑301 (MQ=255)`  
                                                                                                                                                   `gAACTCATGGCAAGCACGTAATGAAGCCTTACAGTTCCGTAATGAGGCTGAGACTTTCAGAAACCAAGC‑GGAGGGCTTCAAGAACGAGTCCAGTACCAACGCTAC‑GAACACAAAGCAGTGGCGCGATGAGACCAAGGGTTTCCGAGACGAAGCCGAGCGGTTCAAGAATACGGCTGGTCAAT‑ACGCTACATCTGCTGGG‑AACTCTGCTTCCGCTGCGCATCAATCTGAGGTAAACGCTGAGAACTCTGCCACAGCATCCGCTAACTCTGCCCATTTGGCAGAACAGCAAGCAGACCGTGCg                                                                                                                                                            >  1:673790/1‑301 (MQ=255)`  
                                                                                                                                                       `acATGGCAAGCACGTAATGAAGCCTTACAGTTCCGTAATGAGGCTGAGACTTTCAGAAACCAAGC‑GGAGGGCTTTAAGAACGAGTCCAGTACCAACGCTAC‑GAACACAAAGCAGTGGCGCGATGAGACCAAGGGTTTCCGATACGAAGCCAAGCGGTTCAAGAATACGGCTGGTCAAT‑ACGCTACATCTGCTGGG‑AACTCTGCTTCCGCTGCGCATCAATCTGAGGTAAACGCTGAGAACTCTGCCACAGCAGCCGCTAACTCTGCTCATTTGGCAGAACAGCAAGCAGACCGTGCAGAAc                                                                                                                                                        <  1:257315/300‑1 (MQ=255)`  
                                                                                                                                                          `tGGCAAGCACGTAATGAAGCCTTACAGTTCCGTAATGAGGCTGAGACTTTCAGAAACCAAGC‑GGAGGGCTTTAAGAACGAGTCCAGTACCAACGCTAC‑GAACACAAAGCAGTGGCGCGATGAGACCAAGGGTTTCCGAGACGAAGCCAAGCGGTTCAAGAATACGGCTGGTCAAT‑ACGCTACATCTGCTGGG‑AACTCTGCTTCCGCTGCGCATCAATCTGAGGTAAACGCTGAGAACTCTGCCACAGCAGCCGCTAACTCTGCTCATTTGGCAGAACAGCAAGCAGACCGTGCGGAACGTg                                                                                                                                                     >  1:203699/1‑301 (MQ=255)`  
                                                                                                                                                              `gagacagTAATGAAGCCTTACAGTTCCGTAATGAGGCTGAGACATTCAGAAACCAAGC‑GGAGGGCTTTAAGAACGAGTCCAGTACCAACGCTAC‑GAACACAAAGCAGTGGCGCGATGAGACCAAGGGTTTCCGAGACGAAGCCAAGCGGTTCAAGAATACGGCTGGTCAAT‑ACGCTACATCTGCTGGG‑AACTCTGCTTCCGCTGCGCATCAATCTGAGGTAAACGCTGAGAACTCTGCCACAGCAGCCGCTAACTCTGCTCATTTGGCAGAACAGCAAGCAGACCGTGCGGAACGTGAGGc                                                                                                                                                 <  1:472657/295‑1 (MQ=255)`  
                                                                                                                                                              `aaGCACGTAATGAAGCCTTACAGTTCCGTAATGAGGCTGAGACTTTCAGAAACCAAGC‑GGAGGGCTTTAAGAACGAGTCCAGTACCAACGCTAC‑GAACACAAAGCAGGGGCGCGATGAGACCAAGGGTTTCCGAGACGAAGCCGAGCGGTTCAAGAATACGGCTGGTCAAT‑ACGCTACATCTGCTGGG‑AACTCTGCTGCCGCTGCGCATCAATCTGAGGTAAACGCTGAGAACTCTGCCACAGCAGCCGCTAACTCTGCTCATTTGGCAGAACAGCAAGCAGACCGTGCGGAACGTGAGGc                                                                                                                                                 <  1:61965/301‑1 (MQ=255)`  
                                                                                                                                                               `aGCACGTAATGAAGCCTTACAGTTCCGTAATGAGGCTGAGACTTTCAGAAACCAAGC‑GGAGGGCTTTAAGAACGAGTCCAGTACCAACGCTAC‑GAACACAAAGCAGTGGCGCGATGAGACCAAGGGTTTCCGAGACGAAGCCAAGCGGTTCAAGAATACGGCTGGTCAAT‑ACGCTACATCTGCTGGG‑AACTCTGCTTCCGCTGCGCATCAATCTGAGGTAAACGCTGAGAACTCTGCCACAGCAGCCGCTAACTCTGCTCATTTGGCAGAACAGCAAGCAGACCGTGCGGAACGTGAGGCa                                                                                                                                                >  1:278611/1‑301 (MQ=255)`  
                                                                                                                                                                `gCACGTAATGAAGCCTTACAGTTCCGTAATGAGGCTGAGACTTTCAGAAACCAAGC‑GGAGGGCTTTAAGAACGAGTCCAGTACCAACGCTAC‑GAACACAAAGCAGTGGCGCGATGAGACCAAGGGTTTCCGAGACGAAGCCAAGCGGTTC‑AGAATACGGCTGGTCAAT‑ACGCTACATCTGCTGGG‑AACTCTGCTTCCGCTGCGCATCAATCTGAGGTAAACGCTGAGAACTCTGCCACAGCAGCCGCTAACTCTGCTCATTTGGCAGAACAGCAAGCAGACCGTGCGGAACGTGAGGCAGa                                                                                                                                              >  1:551820/1‑301 (MQ=255)`  
                                                                                                                                                                 `cACGTAATGAAGCCTTACAGTTCCGTAATGAGGCTGAGACTTTCAGAAACCAAGC‑GGAGGGCTTTAAGAACGAGTCCAGTACCAACGCTAC‑GAACACAAAGCAGTGGCGCGATGAGACCAAGGGTTTCCGAGACGAAGCCGAGCGGTTCAAGAATACGGCTGGTCAAT‑ACGCTACATCTGCTGGG‑AACTCTGCTTCCGCTGCGCATCAATCTGAGGTAAACGCTGAGAACTCTGCCACAGCATCCGCTAACTTTGCTCATTTGGCAGAACAGCAAGCAGACCGTGCGGAACGTGAGGCAGa                                                                                                                                              >  1:603926/1‑301 (MQ=255)`  
                                                                                                                                                                 `cACGTAATGAAGCCTTACAGTTCCGTAATGAGGCTGAGACTTTCAGAAACCAAGC‑GGAGGGCTTTAAGAACGAGTCCAGTACCAACGCTAC‑GAACACAAAGCAGTGGCGCGATGAGACCAAGGGTTTCCGAGACGAAGCCAAGCGGTTCAAGAATACGGCTGGTCAAT‑ACGCTACATCTGCTGGG‑AACTCTGCTTCCGCTGCGCATCAATCTGAGGTAAACGCTGAGAACTCTGCCACAGCAGCCGCTAACTCTGCTCATTTGGCAGAACAGCAAGCAGACCGTGCGGactgtctcttata                                                                                                                                              >  1:498107/1‑289 (MQ=255)`  
                                                                                                                                                                               `ttACAGTTCCGTAATGAGGCTGAGACTTTCAGAAACCAAGC‑GGAGGGCTTTAAGAACGAGTCCAGTACCAACGCTAC‑GAACACAAAGCAGTGGCGCGATGAGACCAAGGGTTTCCGAGACGAAGCCGAGCGGTTCAAGAATACGGCTGGTCAAT‑ACGCTACATCTGCTGGG‑AACTCTGCTTCCGCTGCGCATCAATCTGAGGTAAACGCTGAGAACTCTGCCACAGCATCCGCTAACTATGCTCATTTGGCAGAACAGCAAGCAGACCGTGCGGAACGTGAGGCAGACAAGCTGGAAAAtt                                                                                                                                >  1:625879/1‑301 (MQ=255)`  
                                                                                                                                                                                `tACAGTTCCGTAATGAGGCTGAGACTTTCAGAAACCAAGC‑GGAGGGCTTTAAGAACGAGTCCAGTACCAACGCTAC‑GAACACAAAGCAGTGGCGCGATGAGACCAAGGGTTTCCGAGACGAAGCCGAGCGGTTCAAGAATACGGCTGGTCAAT‑ACGCTACATCTTCTGGG‑AACTCTGCTTCCGCTGCGCATCAATCTGAGGTAAACGCTGAGAACTCTGCCACAGCAGCCGCTAACTCTGCTCATTTGGCAGAACAGCAAGCAGACCGTTCGGAACGTGAGGCAGACAAGCTGGAAAATTa                                                                                                                               <  1:681581/301‑1 (MQ=255)`  
                                                                                                                                                                                 `aagagacagTAATGAGGCTGAGACTTTCAGAAACCAAGC‑GGAGGGCTTTAAGAACGAGTCCAGTACCAACGCTAC‑GAACACAAAGCAGTGGCGCGATGAGACCAAGGGTTTCCGAGACGAAGCCAAGCGGTTCAAGAATACGGCTGGTCAAT‑ACGCTACATCTGCTGGG‑AACTCTGCTTCCGCTGCGCATCAATCTGAGGTAAACGCTGAGAACTCTGCCACAGCATCCGCTAACTCTGCTCATTTGGCAGAACAGCACGCAGACCGTGCGGAATGTGAGGCAGACAAGCTGGAAAATTAc                                                                                                                              <  1:100176/293‑1 (MQ=255)`  
                                                                                                                                                                                    `gTTCCGTAATGAGGCTGAGACTTTCAGAAACCAAGC‑GGAGGGCTTTAAGAACGAGTCCAGTACCAACGCTAC‑GAACACAAAGCAGTGGCGCGATGAGACCAAGGGTTTCCGAGACGAAGCCGAGCGGTTCAAGAATACGGCTGGTCAAT‑ACGCTACATCTGCTGGG‑AACTCTGCTTCCGCTGCGCATCAATCTGAGGTAAACGCTGAGAACTCTGCCACAGCAGCCGCTAACTCTGCTCATTTGGCAGAACAGCAAGCAGACCGTGCGGAACGTGAGGCAGACactgtctcttatacacat                                                                                                                           >  1:210749/1‑284 (MQ=255)`  
                                                                                                                                                                                    `gTTCCGTAATGAGGCTGAGACTTTCAGAAACCAAGC‑GGAGGGCTTTAAGAACGAGTCCAGTACCAACGCTAC‑GAACACAAAGCAGTGGCGCGATGAGACCAAGGGTTTCCGAGACGAAGCCAAGCGGTTCAAGAATACGGCTGGTCAAT‑ACGTTACATCTGCTGGG‑AACTCTGCTTCCGCTGCGCATCAATCTGAGGTAAACGCTGAGAACTCTGCCACATCAGCCGCTAACTCTGCTCATTTGGCAGAACAGCAAGCAGACCGTGCGGAACGTGAGGCAGACAAGCTGGAAAATTACAAt                                                                                                                           >  1:5905/1‑301 (MQ=255)`  
                                                                                                                                                                                    `gTTCCGTAATGAGGCTGAGACTTTCAGAAACCAAGC‑GGAGGGCTTTAAGAACGAGTCCAGTACCAACGCTAC‑GAACACAAAGCAGTGGCGCGATGAGACCAAGGGTTTCCGAGACGAAGCCAAGCGGTTCAAGAATACGGCTGGTCAAT‑ACGCTACATCTGCTGGG‑AACTCTGCTTCCGCTGCGCATCAATCTGAGGTAAACGCTGAGAACTCTGCCACAGCATCCGCTAACTATGCTCATTTGGCAGAACAGCAAGCAGACCGTGCGGAACGTGAGGCAGACAAGCTGGAAAATTACAAt                                                                                                                           >  1:119595/1‑301 (MQ=255)`  
                                                                                                                                                                                      `tCCGTAATGAGGCTGAGACTTTCAGAAACCAAGC‑GGAGGGCTTTAAGAACGAGTCCAGTACCAACGCTAC‑GAACACAAAGCAGTGGCGCGATGAGACCAAGGGTTTCCGAGACGAAGCCAAGCGGTTCAAGAATACGGCTGGTCAAT‑ACGCTACATCTGCTGGG‑AACTCTGCTTCCGCTGCGCATCAATCTGAGGTAAACGCTGAGAACTCTGCCACAGCAGCCGCTAACTCTGCTCATTTGGCAGAACAGCAAGCAGACCGTGCGGAACGTGAGGCAGACAAGCTGG‑AAATTACAATGGa                                                                                                                        >  1:449899/1‑301 (MQ=255)`  
                                                                                                                                                                                            `gtGAGGCTGAGACTTTCAGAAACCAAGC‑AGAGGGCTTTAAGAACGAGTCCAGTAACAACGCTAC‑GAACACAAAGCAGTGGCGCGATGAGACCAAGGGTTTCCGAGACGAAGCCGAGCGGTTCAAGAATACGGCTGGTCAAT‑ACGCTACATCTGCTGGG‑AACTCTGCTTCCGCTGCGCATCAATCTGAGGTAAACGCTGAGAACTCTGCCACAGCATCCGCTAACTTTGCTCATTTGGCAGAACAGCAAGCAGACCGTGCGGAACGTGAGGCAGACAAGCTGGAAAATTACGATGGATTGGc                                                                                                                   >  1:367099/2‑301 (MQ=255)`  
                                                                                                                                                                                             `tGAGGCTGAGACTTTCAGAAACCAAGC‑GGAGGGCTTTAAGAACGAGTCCAGTACCAACGCTAC‑GAACACAAAGCAGTGGCGCGATGAGACCAAGGGTTTCCGAGACGAAGCCGAGCGGTTCAAGAATACGGCTGGTCAAT‑ACGCTACATCTGCTGGG‑AACTCTGCTTCCGCTGCGCATCAATCTGAGGTAAACGCTGAGAACTCTGCCACAGCATCCGCTAACTCTGCTCATTTGGCAGAACAGCACGCAGACCGTGCGGAACGTGAGGCAGACAAGCTGGAAAATTACAATGGATTGGCt                                                                                                                  <  1:729744/301‑1 (MQ=255)`  
                                                                                                                                                                                             `ggAGGATGAGACTTTCAGAAACCAAGC‑GGAGGGCTTTAAGAACGAGTCCAGTACCAACGCTAC‑GAACACAAAGCAGTGGCGCGATGAGACCAAGGGTTTCCGAGACGAAGCCAAGCGGTTCAAGAAGACGGCTGGTAAAT‑ACGCTAAATCCGCTGGG‑AACACTGCTTCCGCTGCGCATCAATCTGAGGGAACCGCTGAAAACGCTGTCACAGCAGCAGCTAACTCTGCTCATGTGGCAGAACAGCACGCAGACCGTGCGGAACGTGAGGCAGACAAGCTGGAAAATTACAATGGATTGGCt                                                                                                                  <  1:124945/300‑1 (MQ=255)`  
                                                                                                                                                                                                  `cTGAGACTTTCAGAAACCAAGC‑GGAGGGCTTTAAGAACGAGTCCAGTACCAACGCTAC‑GAACACAAAGCAGTGGCGCGATGAGACCAAGGGTTTCCGAGACGAAGCCAAGCGGTTCAAGAATACGGCTGGTCAAT‑ACGCTACATATGCTGGG‑AAATCTGCTTCCGCTGCGCATCAATCTGAGGTAAACGCTGAGAACTCTGCCACAGCAGCCGCTAACTCTGCTCAGTTGGCAGAACAGCAAGCAGACCGTGCGGAACGTGAGGCAGACAAGCTGGAAAATTACAATGGATTGGCTGGTGc                                                                                                             <  1:374877/301‑1 (MQ=255)`  
                                                                                                                                                                                                      `gaCTTTCAGAAACCAAGC‑GGAGGGATTTAAGAACGAGTCCAGTACCAACGCTAC‑GAACACAAAGCAGTGGCGCGATGAGACCAAGGGTTTCCGAGACGATGCCAAGCGGTTCAAGAATACGGCTGGTCAAT‑ACGCTACATCTGCTGGG‑AACTCTGCTTCCGCTGCGCATCAATCTGAGGTAAACGCTGAGAACTCTGCCACAGCAGCCGCTAACTCTGCTCATTTGGCAGAACAGCAAGCAGACCGTGCGGAACGTGAGGCAGACAAGCTGGAAAATTACAATGGATTGGCTGGTGCAAtt                                                                                                         >  1:344584/1‑301 (MQ=255)`  
                                                                                                                                                                                                       `aCTTTCAGAAACCAAGG‑GGAGGGCTGTAAGAACGAATCCAGTACCAACGCTAC‑GAACACAAAGCAGTGACGGAATGAGACCAAGTGTTTCCGAGACGAAGCCAAGCGGTTCAAGAATACGGCTGGTCAAT‑ACGCTACATCAGCTGGG‑AACTCTGCTTCCGTTGCGTATCAATCTGAGGTAAACGCTGAGAACTCGGCAACAGCAGCCTCTAACTCTGCTCATTTGGCAGAACAGAAAGCAGACCGTGCGGAACGTGAGGCAGACAAGCTGGAAAATTACAATGGATTGGCTGGTGCAATTg                                                                                                        <  1:188105/301‑1 (MQ=255)`  
                                                                                                                                                                                                          `ttCAGAAACCAAGC‑GGAGGGCTTTAAGAACGAGTCCAGTACCAACGCTAC‑GAACACAAAGCAGTGGCGCGATGAGACCAAGGGTTTCCGAGACGAAGCCGAGCGGTTCAAGAATACGGCTGGTCAAT‑ACGCTACATCTGCTGGG‑AACTCTGCTTCCGCTGCGCATCAATCTGAGGTAAACGCTGAGAACTCTGCCACAGCATCCGCTAACTCTGCTCATTTGGCAGAACAGCAAGCAGACCGTGCGGAACGTGAGGCAGACAAGCTGGAAAATTACAATGGATTGGCTGGTGCCATTGATa                                                                                                     >  1:46583/1‑301 (MQ=255)`  
                                                                                                                                                                                                             `aGAAACCAAGC‑GGAGGGCTTTAAGAACGAGTCCAGTACCAACGCTAC‑GAACACAAAGCAGTGGCGCGATGAGACCAAGGGTTTCCGAGACGAAGCCGAGCGGTTCAAGAATACGGCTGGTCAAT‑ACGCTACATCTGCTGGG‑AACTCTGCTTCCGCTGCGCATCAATCTGAGGTAAACTCTGAGAACTCTGCCACAGCAGCCGCTAACTCTGCTCATTTGGCAGAACAGCAAGCAGACCGTGCGGAACGTGAGGCAGACAAGCTGGAAAATTACAATGGATTGGCTGGTGCAATTGATAAgg                                                                                                  <  1:575824/301‑1 (MQ=255)`  
                                                                                                                                                                                                                             `ggCTTTAAGAACGAGTCCAGTACCAACGCTAC‑GAACACAAAGCAGTGGCGCGATGAGACCAAGGGTTTCCGAGACGAAGCCAAGCGGTTCAAGAATACGGCTGGTCAAT‑ACGCTACATCTGCTGGG‑AACTCTGCTTCCGCTGCGCATCAATCTGAGGTAAACGCTGAGAACTCTGCCACAGCAGCCGCTAACTCTGCTCATTTGGCAGAACAGCAAGCAGACCGTGCGGAACGTGAGGCAGACAAGCTGGAAAATTACAATGGATTGGCTGGTGCAATTGATAAGGTAGATGGactgtctt                                                                                   >  1:166079/1‑294 (MQ=255)`  
                                                                                                                                                                                                                              `gCTTTAAGAACGATTCCAGTACCAACGCTAC‑GAACACAAAGCAGTGGCGCGATGAGACCAAGGGTTTCCGAGACGAAGCCGAGCGGTTCAAGAATACGGCTGGTCAAT‑ACGCTACATCTGCTGGG‑AACTCTGCTTCCGCTGCGCATCAATCTGAGGTAAACGCTGAGAACTCTGCCACAGCATCCGCTAACTCTGCTCATTTGGCAGAACAGCACGCAGACCGTGCGGAACATGAGGCAGACAAGCTGGAAAATTACAATGGATTGGCTGGTGCAATTGATAAGGTAGATGGAACCAAtgg                                                                                  >  1:263459/1‑300 (MQ=255)`  
                                                                                                                                                                                                                              `gCTTTAAGAACGAGTCCAGTACCAACGCTAC‑GAACACAAAGCAGTGGCGCGATGAGACCAAGGGTTTCCGAGACGAAGCCAAGCGGTTCAAGAATACGGCTGGTCAAT‑ACGCTACATCTGCTGGG‑AACTCTGCTTCCGCTGCGCATCAATCTGAGGTAAACGCTGAGAACTCTGCCACAGCATCCGCTAACTATGCTCATTTGGCAGAACAGCAAGCAGACCGTGCGGAACGTGAGGCAGACAAGCTGGAAAATTACAATGGATTGGCTGGTGCAATTGATAAGGTAGATGGactttctct                                                                                  >  1:770855/1‑293 (MQ=255)`  
                                                                                                                                                                                                                               `cTTTAAGAACGAGTCCAGTACCAACGCTAC‑GAACACAAAGCAGTGGCGCGATGAGACCAAGGGTTTCCGAGACGAAGCCGAGCGGTTCAAGAATACGGCTGGTCAAT‑ACGCTACATCTGCTGGG‑AACTCTGCTTCCGCTGCGCATCAATCTGAGGTAAACGCTGAGAACTCTGCCACAGCATCCGCTAACTTTGCTCATTTGGCAGAACAGCAAGCAGACCGTGCGGAACGTGAGGCAGACAAGCTGGAAAATTACAATGGATTGGCTGGTGCAATTGATAAGGTAGATGGAAcctgtctc                                                                                 >  1:219494/1‑295 (MQ=255)`  
                                                                                                                                                                                                                               `cTTTAAGAACGAGTCCAGTACCAACGCTAC‑GAACACAAAGCAGTGGCGCGATGAGACCAAGGGTTTCCGAGACGAAGCCGAGCGGTTCAAGAATACGGCTGGTCAAT‑ACGCTACATCTGCTGGG‑AACTCTGCTTCCGCTGCGCATCAATCTGAGGTAAACGCTGAGAACTCTGCCACAGCATCCGCTAACTCTGCTCATTTGGCAGAACAGCAAGCAGACCGTGCGGAACGTGAGGCAGACAAGCTGGAAAATTACAATGGATTGGCTGGTGCAATTGATAAGGTAGATGGAACCAAtgtg                                                                                 >  1:152411/1‑301 (MQ=255)`  
                                                                                                                                                                                                                                  `aaaGAACGAGTCCAGTACCAACGCTAC‑GAACACAAAGCAGTGGCGCGATGAGACCAAGGGTTTCCGAGACGAAGCCGAGCGGTTCAAGAATACGGCTGGTCAAT‑ACGCTACATCTGCTGGG‑AACTCTGCTTCCGCTGCGCATCAATCTGAGGTAAACGCTGAGAACTCTGCCACAGCATCCGCTAACTATGCTCATTTGGCAGAACAGCAAGCAGACCGTGCGGAACGTGAGGCAGACAAGATGGAAAATTACAATGGATTGGCTGGTGCAATTGATAAGGTAGATGGAACAAATGTGTAc                                                                              <  1:784662/300‑1 (MQ=255)`  
                                                                                                                                                                                                                                       `accaggccAGTACGAACGCTAG‑GAACAAAAAGAAGTGGCGCGATGAGACCAAGGGTTTCCGAGACGAAGCCAAGCGGTTCAAGAATACGGCTGGTCAAT‑ACGCTACATCTGCTGGG‑AACTCTGCTTCCGCTGCGCATCAATCTGAGGTAAACGCTGAGAACTCTGCCACAGCAGCCGCTAACTCTGCTCATTTGGCAGAACAGCAAGCAGACCGTGCGGAACGTGAGGCAGACAAGCTGGAAAATTACAATGGATTGGCTGGTGCAATTGATAAGGTAGATGGAACCAATGTGTACTggaa                                                                         <  1:405416/295‑1 (MQ=255)`  
                                                                                                                                                                                                                                       `aCGAGTCCAGTACCAACGCTAC‑GAACACAAAGCAGTGGCGCGATGAGACCAAGGGTTTCCGAGACGAAGCCAAGCGATTCAAGAATACGGCTGGTCAAT‑ACGCTACATCTGCTGGG‑AACTCTGCTTCCGCTGCGCATCAATCTGAGGTAAACGCTGAGAACTCTGCCACAGCAGCCGCTAACTCTGCTCATTTGGCAGAACAGCAAGCAGACCGTGCGGAACGTGAGGCAGACAAGCTGGAAAATTACAATGGATTGGCTGGTGCAATTGATAAGGTAGATGGAACCAATGTGTACTggaa                                                                         <  1:412745/301‑1 (MQ=255)`  
                                                                                                                                                                                                                                           `gTCCAGTACCAACGCTAC‑GAACACAAAGCAGTGGCGCGATGAGACCAAGGGTTTCCGAGACGAAGCCAAGCGGCTCAAGAATACGGCTGGTCAAT‑ACGCTACATCTGCTGGG‑AACTCTGCTTCCGCTGCGCATCAATCTGAGGTAAACGCTGAGAACTCTGCCACAGCAGCCGCTAACTCTGCTCATTTGGCAGAACAGCAAGCAGACCGTGCGGACCGTGAGGCAGACAAGCTGGAAAATTACAATGGATTGGCTGGTGCAATTGATAAGGTAGATGGAAACAATGTGTACTggaaagga                                                                     >  1:49019/1‑301 (MQ=255)`  
                                                                                                                                                                                                                                             `ccAGTACCAACGCTAC‑GAACACAAAGCAGTGGCGCGATGAGACCAAGGGTTTCCGAGACGAAGCCAAGCGGTTCAAGAATACGGCTGGTCAAT‑ACGCTACATCTGCTGGG‑AACTCTGCTTCCGCTGCGCATCAATCTGAGGTAAACGCTGAGAACTCTGCCACAGCAGCCGCTAACTCTGCTCATTTGGCAGAACAGCAAGCAGACCGTGCGGAACGTGAGGCATACAAGCTGGAAAATTACAATGGATTGGCTGGTGCAATTGATAAGGTAGATGGAACCAATGTGTACTggaaaggaaa                                                                   <  1:815429/301‑1 (MQ=255)`  
                                                                                                                                                                                                                                               `aGTACCAACGCTAC‑GAACACAAAGCAGTGGCGCGATGAGACCAAGGGTTTCCGAGACGAAGCCAAGCGGTTCAAGGATACGGCTGGTCAAT‑ACGCTACATCTGCTGGG‑AACTCTGCTTCCGCTGCGCATCAATCTGAGGTAAACGCTGAGAACTCTGCCACAGCAGCCGCTAACTCTGCTCATTTGGCAGAACAGCAAGCAGACCGTGCGGAACGTGAGGCAGACAAGCTGGAAAATTACAATGGATTGGCTGGTGCAATTGATAAGGTAGATGGAACCAATGTGTACTGGAAAGGAAata                                                                 <  1:399220/301‑1 (MQ=255)`  
                                                                                                                                                                                                                                                `gTACCAACGCTAC‑GAACACAAAGCAGTGGCGCGATGAGACCAAGGGTTTCCGAGACGAAGCCGAGCGGTTCAAGAATACGGCTGGTCAAT‑ACGCTACATCTGCTGGG‑AACTCTGCTTCCGCTGCGCATCAATCTGAGGTAAACGCTGAGAACTCTGCCACAGCATCCGCTAACTCTGCTCATTTGGCAGAACAGCAAGCAGACCGTGCGGAACGTGAGGCAGACAAGCTGGAAAATTACAATGGATTGGCTGGTGCAATTGATAAGGTAGATGGAACCAATGTGTACTGGAAAGGAAatat                                                                >  1:180768/1‑301 (MQ=255)`  
                                                                                                                                                                                                                                                 `tACCAACGCTAC‑GAACACAAAGCAGTGGCGCGATGAGACCAAGGGTTTCCGACACGAAGCCAAGCGGTTCAAGAATACGGCTGGTCAAT‑ATGCAACATCTGCTGGG‑AACTCTGATTCCGCTGCGCAAAAACCTGAGGTTAACGCTGAGACCTCTGCAACAACAGCCGCTAACTCTGCTCATTTGGAAGAACAGCAAGCAGACCGTGCGGAACGTGAGGCAGACAAGCTGGAAAATAACAATGGATTGGCTGGAGCAATTGATAAGGTAGAGAGAACGAATGTGTACTGGAAAGGAAATAtt                                                               >  1:567524/1‑301 (MQ=255)`  
                                                                                                                                                                                                                                                         `cTAC‑GAACACAAAGCAGTGGCGCGATGAGACCAAGGGTTTCCGAGACGAAGCCAAGCGGTTCAAGAATACGGCTGGTCAAT‑ACGCTACATCTGCTGGG‑AACTCTGCTTCCGCTGCGCATCAATCTGAGGTAAACGCTAAGAACTCTGCCACAGCAGCCGCTAACTCTGCTCATTTGGCAGAACAGCAAGCAGACCGTGCGGAACGTGAGGCAGACAAGCTGGAAAATTACAATGGATTGGCTGGTGCAATTGATAAGGTAGATGGAACCAATGTGTACTGGAAAGGAAATATTCACGCTaa                                                       >  1:330600/1‑301 (MQ=255)`  
                                                                                                                                                                                                                                                         `cTAC‑GAACACAAAGCAGTGGCGCGATGAGACCAAGGGTTTCCGAGACGAAGCCAAGCGGCTCAAGAATACGGCTGGGCAAT‑ACGCTACATCTGCTGGG‑AACTCTGCTTCCGCTGCGCATCAATCTGAGGTAAACGCAGAGAACTCTGCAACAGCATCCGCTAACTATGCTCATTTGGCAGAACAGCAAGCAGACCGTGCGGAACGTGAGGCAGACAAGCTGGAAAATTACAATGGATTGGCTGGTGCAATTGATAAGGTAGATGGAACCAATGTGTACTGGAAAGGAAATATTCACGCGaa                                                       >  1:73729/1‑301 (MQ=255)`  
                                                                                                                                                                                                                                                         `cTAC‑GAACACAAAGCAGTGGCGAGATGAGACCAAGGGTTTCCGAGACGAAGCCGAGCGGTTCAAGAATACGGCTGGTCAAT‑ACGCTACATCTGCTGGG‑AACTCTGCTTCCGCTGCGCATCAATCTGAGGTAAACGCTGAGAACTCTGCCACAGCATCCGCTAACTTTGCTCATTTGGCAGAACAGCAAGCAGACCGTGCGGAACGTGAGGCAGACAAGCTGGAAAATTACAATGGATTGGCTGGTGCAATTGATAAGGTAGATGGAACCAATGTGTACTGGAAAGGAAATATTCACGCTaa                                                       >  1:48204/1‑301 (MQ=255)`  
                                                                                                                                                                                                                                                           `aC‑GAACACAAAGCAGTGGCGCGATGAGACCAAGGGTCTCAGAGACGAAGCCGAGAGGTTCAAGAATACGGCTGGTCAAT‑ACGCTACATCTGCTGGG‑AACTCTGCTTCCGATGCGCATCAATCTGAGGGAAACGCTGAGAACTCTGCCACAGCAGCCGCTAACTCTGCTAATTTGGCAGAACAGAAAGCAGACCGTGCGGAACGTGAGGAAGACAAGCTGGAAAATTACAATGGATTGGCTGGTGCAATTGATAAGGTAGATGGAAACAATGTGTACTGGAAAGGAAATATTAACGCTAACg                                                     >  1:633961/1‑301 (MQ=255)`  
                                                                                                                                                                                                                                                                   `caAAGCAGTGGCGCGATGAGACCAAGGGTTTCCGAGACGAAGCCGAGCGGTTCAAGAATACGGCTGGTCAAT‑ACGCTACATCTGCTGGG‑ACCTCTTCTTCCGCTGCGCATCAATCTGAGGTACACGCTGAGAACTCTGCCCCATCATCCGCTAACTATGCTCCTTTGGCAGAACAGCAGGCCGACCGTGCGGCCCGTGAGGCCGACATGCTGGACAATTACAATGGATTGGAGGGTGCAATTGAGAAGGTAGCTGGAACCAATGTGTACTGGAAATGAAATATTCACTCTCACGGGCGCCt                                              >  1:196799/1‑301 (MQ=255)`  
                                                                                                                                                                                                                                                                   `caAAGCAGTGGCGCGATGAGACCAAGGGTTTCCGAGACGAAGCCGAGCGGTTCAAGAATACGGCTGGTCAAT‑ACGCTACATCTGCTGGG‑AACTCTGCTTCCGCTGCGCATCAATCTGAGGTAAACGCTGAGAACTCTGCCACAGCATCCGCTAACTATGCTCATTTGGCAGAACAGCAAGCAGACCGTGCGGAACGTGAGGCAGACAAGCCGGAAAATTACAATGGATTGGCTGGTGCAATTGATAAGGTAGATGGAACCAATGTGTACTGGAAAGGAAATATTCACGCTAACGGGCGCCt                                              >  1:702940/1‑301 (MQ=255)`  
                                                                                                                                                                                                                                                                       `gCAGTGGCGCTATGAGACCAAGGGTTTCCGAGACGAAGCCGAGCGGTTCAAGAATACGGCTGGTCAAT‑ACGCTACATCTGCTGGG‑AACTCTGCTTCCGCTGCGCATCAATCTGAGGAAAACGCTGAGAACTCTGCCACAGCATCCGCTAACTATGCTCATTTGGCAGAACAGCAAGCAGACCGTGCGGAACGTGAGGCAGACAAGCTGGAAAATTACAATGGATTGGCTGGTGCAATTGATAAGGTAGATGGAACCAATGTGTACTGGAAAGGAAATATTCACGCTAACGGGCGCCTTTaa                                          >  1:436924/1‑300 (MQ=255)`  
                                                                                                                                                                                                                                                                       `gCAGTGGCGCGATGAGACCAAGGGTTTCCGAGACGAAGCCAAGCGGTTCAAGAATACGGCTGGTCAAT‑ACGCTACATCTGCTGGG‑AACTCTGCTTCCGCTGCGCATCAATCTGAGGTAAACGCTGAGAACTCTGCCACAGCATCCGCTAACTATGCTCATTTGGCAGAACAGCAAGCAGACCGTGCGGAACGTGCGGCAGACAAGCTGGAAAATTACAATGGATTGGCTGGTGCAATTGATAAGGTAGATGGAACTAATGTGTACTGGAAAGGAAATATTCACGCTAACGGGCGCCTTTAc                                          >  1:666422/1‑301 (MQ=255)`  
                                                                                                                                                                                                                                                                        `gaGTGGAGCGATGAGACCAAGGGGTTCCGAGACGAAGCCAAGCGGTTCAAGAATACGGCTGGTCAAT‑ACGCTACATCTGCTGGG‑AACCCTGCTTCCGCTGCGCATCAATCTGAGGTAAACGCTGAGAACTCTGCCACAGCAGCCGCTAACTCTGCTCATTTGGCAGAACAGCAAGCAGACCGTGCGGAACGTGAGGCAGACAAGCTGGAAAATTACAATGGATTGGCTGGTGCAATTGATAAGGTAGATGGAACCAATGTGTACTGGAAAGGAAATATTCACGCTAACGGGCGCCTTTAcc                                         <  1:173305/300‑2 (MQ=255)`  
                                                                                                                                                                                                                                                                          `gTGGCGCGATGAGACCAAGGGTTTCCGAGACGAAGCCGAGCGGTTCAAGAATACGGCTGGTCAAT‑ACGCTACATCTGCTGGG‑AACTCTGCTTCCGCTGCGCATCAATCTGAGGTAAACGCTGAGAACTCTGCCACAGCATCCGCTAACTCTGCTCATTTGGCAGAACAGCAAGCAGACCGTGCGGAACGTGAGGCAGACAAGCTGGAAAATTACAATGGATTGGCTGGTGCAATTGATAAGGTAGATGGAACCAATGTGTACTGGAAAGGAAATATTCACGCTAACGGGCGCCTTTACCTg                                       >  1:131737/1‑301 (MQ=255)`  
                                                                                                                                                                                                                                                                            `ggCGCGATGAGACCAAGGGTTTCCGAGACGAAGCCAAGCGGTTCAAGAATACGGCTGGTCAAT‑ACGCTACATCTGCTAGG‑AACTCTGCTTCCGCTGCGCATCAATCTGAGGTAAACTCTGAGAACTCTGCCACAGCAGCCGCTAACTCTGCTCATTTGGCAGAACAGCAAGCAGACCGTGCGGAACGTGAGGCAGACAAGCTGGAAAATTACAATGGATTGGCTGGTGCAATTGATAAGGTAGATGGAACCAATGTGTACTGGAAAGGAAATATTCACGCTAACGGGCGCCTTTACATGAc                                     >  1:612700/1‑301 (MQ=255)`  
                                                                                                                                                                                                                                                                                 `gATGAGACCAAGGGTTTCCGAGACGAAGCCAAGCGGTTCAAGAATACGGCTGGTCAAT‑ACGCTACATCTGCTGGG‑AACTCTGCTTCCGCTGCGCATCAATCTGAGGTAAACGCTGAGAACTCTGCCACAGCAGCCGCTAACTCTGCTCATTTGGCAGAACAGCAAGCAGACCGTGCGGAACGTGAGGCAGACAAGCTGGAAAATTACAATGGATTGGCTGGTGCACTTGATAAGGTAGATGGAACCAATGTGTACTGGAAAGGAAATATTCACGCTAACGGGCGCCTTTACATGACCACaa                                >  1:658595/1‑301 (MQ=255)`  
                                                                                                                                                                                                                                                                                              `gTTTCCGAGACGAAGCCGAGCGGTTCAAGAATACGGCTGGTCAAT‑ACGCTACATCTGCTGGG‑AACTCTGATTCCGCTGCGCATCAATCTGAGGTAAACGCTGAGAACTCTGCCACAGCAGCCGCTAACTCTGCTCATTTGGCAGAACAGCAAGCAGACCGTGCGGAACGTGAGGCAGACAAGCTGGAAAATTACAATGGATTGGCTGGTGCAATTGATAAGGTAGATGGAACAAATGTGTACTGGAAAGGAAATATTCACGCTAACGGGCGCCTTTACATGACCACAAACGGTTTTGACtg                   <  1:596771/301‑1 (MQ=255)`  
                                                                                                                                                                                                                                                                                                      `gaCGAAGCCAAGCGGTTCAAGGATACGGCTGGTCAAT‑ACGCTACATCTGCTGGG‑AACTCTGCTTCCGCTGCGCATCAATCTGAGGTAAACGCTGAGAACTCTGCCACAGCATCCGCTAACTCTGCTCATTTGGCAGAACAGCAAGCAGACCGTGCGGAACGTGCGGCAGACAAGCTGGAAAATTACAATGGATTGGCTGGTGCAATTGATAAGGTAGATGGAACCAATGTGTACTGGAAAGGAAATATTCACGCTAACGGGCGCCTTTACATGACCACAAACGGTTTTGACTGTGGCCAGt           >  1:538143/1‑301 (MQ=255)`  
                                                                                                                                                                                                                                                                                                          `aaGCCAAGCGGTTCAAGAATACGGCTGGTCAAT‑ACGCTACATCTGCTGGG‑AACTCTGCTTCCGCTGCGCATCAATCTGAGGTAAACGCTGAGAACTCTGCCACAGCATCCGCTAACTATGCTCATTTGGCAGAACAGCAAGCAGACCGTGCGGAACGTGCGGCAGACAAGCTGGAAAATTACAATGGATTGGCTGGTGCAATTGATAAGGTAGATGGAACCAATGTGTACTGGAAAGGAAATATTCACGCTAACGGGCGCCTTTACATGACCACAAACGGTTTTGACTGTGGCCAGTATCa       >  1:640668/1‑301 (MQ=255)`  
                                                                                                                                                                                                                                                                                                            `gCCAAGCGGTTCAAGAATATGGCTGGTCAAT‑ACGCTACATCTGCTGGG‑AACTCTGCTTCCGCTGCGCATCAATCTGAGGTAAACGCTGAGAACTCTGCCAAAGCATCCGCTAACTCTGCTCATTTGGCAGAACAGCAAGCAGACCGTGCGGAACGTGAGGCAGACAAGCTGGAAAATTACAATGGATTGGCTGGTGCAATTGATAAGGTAGATGGAACCAATGTGTACTGGAAAGGAAATATTCACGCTAACGGGCGCCTTTACCTGACCACAAACGGTTTTGACTGTGGCCAGTATCAAc     <  1:323615/301‑1 (MQ=255)`  
                                                                                                                                                                                                                                                                                                              `cAAGCGGTTCAAGAATACGGCTGGTCAAT‑ACGCTACATCTTCTGGG‑AACTCTGCTTCCGCTGCGCATCAATCTGAGGTAAACGCTGAGAACTCTGCCACAGCAGCCGCTAACTCTGCTCATTTGGCAGAACAGCAAGCAGACCGTGCGGAACGTGAGGCAGACAAGCTGGAAAATTACAATGGATTGGCTGGTGCAATTGATAAGGTAGATGGAACCAATGTGTACTGGAAAGGAAATATTCACGCTAACGGGCGCCTTTACCTGACCACAAACGGTTTTGACTGTGGCCAGTATCAACAg   <  1:145838/301‑1 (MQ=255)`  

                                                                                                                                                                                                                                                                                                               `|`                                                                                                                                                                                                                                                                                                                
`GAAGAGGCCCGTGACCTCACTACGGATACTATCGGTGTCAATAACGATGGTCACTTGGATGCTCGTGGTCGTCGAATTGTGAACCTAGCGAACGCCGTGGATGACCGCGATGCT‑GTTCCGTTTGGTCAACTAAAGACCATGAACCAGAACTCATGGCAAGCACGTAATGAAGCCTTACAGTTCCGTAATGAGGCTGAGACTTTCAGAAACCAAGC‑GGAGGGCTTTAAGAACGAGTCCAGTACCAACGCTAC‑GAACACAAAGCAGTGGCGCGATGAGACCAAGGGTTTCCGAGACGAAGCCAAGCGGTTCAAGAATACGGCTGGTCAAT‑ACGCTACATCTGCTGGG‑AACTCTGCTTCCGCTGCGCATCAATCTGAGGTAAACGCTGAGAACTCTGCCACAGCATCCGCTAACTCTGCTCATTTGGCAGAACAGCAAGCAGACCGTGCGGAACGTGAGGCAGACAAGCTGGAAAATTACAATGGATTGGCTGGTGCAATTGATAAGGTAGATGGAACCAATGTGTACTGGAAAGGAAATATTCACGCTAACGGGCGCCTTTACATGACCACAAACGGTTTTGACTGTGGCCAGTATCAACAGT  >  NC_001604.1/34954‑35554`

  

Alignment Legend

Aligned base mismatch/match (shaded by quality score): `ATCG/ATCG` `< 3 ≤` `ATCG/ATCG` `< 10 ≤` `ATCG/ATCG` `< 22 ≤` `ATCG/ATCG` `< 35 ≤` `ATCG/ATCG` `< 38 ≤` `ATCG/ATCG`

Unaligned base: `atcg`    Masked matching base: `atcg`    Alignment gap: `‑`    Deleted base: `‑`
